# Pattern of skin diseases amongst children attending a dermatology clinic in Lagos, Nigeria

**DOI:** 10.11604/pamj.2018.29.162.14503

**Published:** 2018-03-19

**Authors:** Olusola Ayanlowo, Oluwaseun Puddicombe, Shakirat Gold-Olufadi

**Affiliations:** 1 Department of Medicine, College of Medicine University of Lagos, Idi-araba, Lagos, Nigeria; 2 Massey Street Children’s Hospital, Lagos, Nigeria

**Keywords:** Prevalence, skin diseases, children, dermatology

## Abstract

**Introduction:**

Skin diseases constitute a major health problem affecting a large proportion of the population including children causing distress and disability. This study aimed to document the spectrum and frequency of skin diseases of children who attended the dermatology outpatient clinic of the Lagos University Teaching Hospital (LUTH) Lagos, Nigeria.

**Methods:**

A cross-sectional study of children (18 years old and below) who attended the dermatology clinic between January 2004 and December 2016. Data obtained from the medical records of the patients included age, gender, clinical features, laboratory features and diagnosis. Skin diseases were classified into various groups.

**Results:**

There were 6373 children included in the study with a male to female ratio of 1:1.13. The most common disease categories were infections 1795 (26.1%), Eczematous conditions 1711 (24.9%), Infestations 936 (13.6%), papulosquamous disorders 547 (8.0%) and Bullous disorders 254 (3.7%). With respect to individual skin diseases, atopic dermatitis (AD) 1042 (15.1%) was the most common skin condition followed by papular urticaria 705 (10.2%) and tinea capitis 554 (8.1%). Infections were most common among infants and adolescents. Acneiform eruptions were common among adolescents while eczematous disorders were most common among children less than 5 years.

**Conclusion:**

The study highlights the common dermatoses seen in children in a specialized dermatology clinic in a developing country such as Nigeria. Most of the skin diseases observed can be controlled by proper environmental sanitation, adequate nutrition, reducing overcrowding, and promoting good health-seeking behavior among parents and caregivers. Information obtained from the study may guide training in dermatology especially among pediatricians.

## Introduction

Skin diseases constitute a major health problem affecting a large proportion of the population causing distress and disability [1]. They represent between 6% and 24% of general paediatric consultations in sub-Saharan Africa with infectious diseases reported as the most common diagnoses made amongst children and adolescents.[2-4]. Despite the high frequency of certain skin diseases in developing countries, they have not been regarded as significant health problem in the development of public health strategies in Nigeria. Some skin disorders are exclusive to childhood, while others are found across all age groups but may differ in manifestation and treatment [5]. The pattern of skin disease in any country is affected by ecological, environmental, racial and social factors as well as literacy levels [4-7]. In Nigeria, recognized predisposing factors to skin disorders in children include poor personal hygiene, low parental level of education, overcrowded living conditions and low socioeconomic status of parents [6-9]. Prevalence studies from the community, primary health centers, dermatology clinics and general outpatient clinics have indicated differences in the distribution of skin diseases. [4, 5, 7-11]. Community-based studies show a high burden of dermatophytosis among school children in both urban and rural areas in Nigeria, [8-10,12] while dermatology clinics have reported a higher incidence of eczematous disorders such as atopic dermatitis [11, 13, 14]. In developing countries, infective disorders mainly pyoderma and scabies have been reported as the major causes for visits among children evaluated in primary health care facilities [1, 4]. In Nigeria, children and adults with skin diseases are managed by physicians in specialty clinics [5, 6] as Paediatric dermatology is an evolving subspecialty [5, 6]. Skin diseases in children require a separate approach from adults because of differences in clinical presentation, treatment and prognosis. As such, there is a need to have accurate knowledge of the skin diseases affecting children. Although hospital-based studies can be affected by factors such as health-seeking behavior, accessibility to healthcare and socioeconomic factors, information obtained from such studies can provide data on the trends of skin disease. Therefore, a better understanding of the disease burden will provide information for health planning. This will go a long way to improving management and prevention of skin disorders. The aim of the study was to document the spectrum and frequency of skin diseases in various age groups of children who attended the dermatology outpatient clinic over a 13-year period.

## Methods

This was a retrospective study which reviewed patients in the paediatric age group (18 years old and below) who presented at the dermatology outpatient clinic of the Lagos University Teaching Hospital (LUTH) Lagos, Nigeria between January 2004 and December 2016, a thirteen-year period. The dermatology clinic of the Lagos University Teaching Hospital (LUTH) Lagos, Nigeria is a tertiary referral center with patients sent from primary health centers and secondary level hospitals within and outside Lagos state. The dermatology unit cares for patients of all age groups since there is no dedicated clinic for paediatric patients. Referrals are also received from paediatric general outpatient clinics, wards and the children emergency department. The clinic attends to between 2000 and 2,500 new cases every year with about a third of these being children. Data of children aged 18 or lower who presented with skin problems from January 2004 - December 2016 were extracted from the clinic register and recorded on Microsoft Excel spreadsheet. Data obtained from the medical records of the patients included age, gender, clinical and laboratory features and diagnosis. The children were stratified into the following age groups: neonates and infants (<2 years) pre-school age (between 2 to 5 years), school age (6 to 12 years) and adolescents (13 to 18 years).

**Ethical clearance:** The study protocol was approved by the Health Research Ethics Committee of the Lagos University Teaching Hospital (LUTH) Lagos, Nigeria (ADM/DCST/HREC/APP/1252). Privacy and confidentiality was ensured by avoiding the use of sensitive information and real names in the proforma for data collection.

**Statistical Analysis:** Data analysis was performed using the software SPSS (Statistical Package for Social Sciences, Chicago, IL, USA) Version 16.0. Frequency tables were used to describe the categorical variables.

## Results

**Figure 1:**
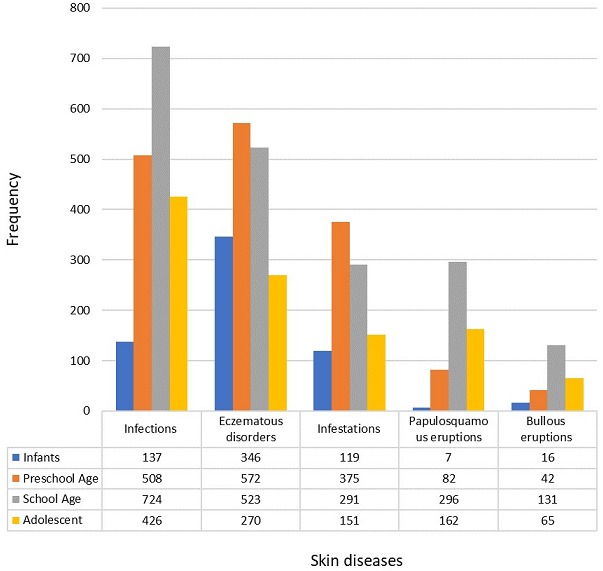
Most common skin disorders by age group

Between January 2004 and December 2016 (study period), a total of 27,025 patients were seen at the dermatology outpatient clinic. The total number of patients aged 18 years and below was 6,373 (23.6%). Out of the 6,373 children who presented, 209 patients did not have clear diagnoses, hence their information was excluded. A total of 6871 diagnoses were made from 6164 patients as some had multiple diagnoses. In the study population, 5646 (91.6%) had one diagnosis, 507 (8.2%) patients had two diagnoses and 9 (0.2%) patients had three diagnoses. The age range was between 4 days and 18 years. The male to female ratio was 1:1.13. Patients were grouped into infants (less than 2 years), preschool age (2 to 6 years), school age (7 to 12 years) and adolescents (13 to 18 years) Table 1. Table 2 shows the spectrum and frequency of skin disorders according to age groups. The five most frequently diagnosed skin conditions include infections, eczematous conditions, infestations, papulosquamous disorders and bullous eruptions in descending order. The least common diseases were xerodermas, granulomatous eruptions and albinism. Fungal infections were the most commonly diagnosed skin infections seen in 1120 (16.3%) patients. Figure 1 describes the prevalence of the most common diseases by age groups. Infectious skin diseases were the most common skin diagnoses observed in preschool and school age children. Among infants and preschoolers, eczematous conditions were most common followed by infections and infestations. In Table 3, Tinea capitis was the most common type of dermatophyte infection in 554 children accounting for about half of those with fungal infections, followed by tinea pedis and tinea corporis. Eighty-four patients (1.4%) with fungal infections also had atopic dermatitis. Candida infection occurred in 114 patients (1.7%) while Pityriasis versicolor occurred in 253 patients (3.7%). Deep mycosis was found in four patients (0.1%) with background immune suppression. Verrucae vulgaris (common warts) was the most frequent viral skin infection followed by molluscum contagiosum and epidermodysplasia verruciformis. Impetigo, mycobaterial infections and periporitis were the most common bacterial infections in descending order. In Table 4, Atopic Dermatitis was the most common presentation of eczematous dermatitis found in 1042 children (15.1%), followed by contact dermatitis (170; 2.5%) while ichthyosis was the most common xerotic disorder. Systemic Lupus Erythematosus was the most documented connective tissue disorder while Neurofibromatosis was the most frequent genodermatosis. Table 5 shows the frequency of papulosquamous eruptions and bullous disorders. Pityriasis rosea, lichenoid eruptions and psoriasis were the most common papulosquamous disorders. Lichen planus was the most common of the lichenoid eruptions. Alopecia areata was the most common type of alopecia observed while Hemangioma was the most common benign skin tumor.

**Table 1:** Demographic characteristics of pediatric patients seen at LUTH Skin Clinic

Characteristics	Number	Percent
**Sex**		
Female	3,385	53.1
Male	2,988	46.9
**Age Groups**		
Infants (< 2years)	674	10.6
Preschool (2 -5 years)	1,755	27.5
School age (7-12 years)	2,253	35.4
Adolescents (13 – 18 years)	1,691	26.5
Male: female ratio	1:1.13	
Mean Age (years) ± Standard Deviation	8.31 ± 5.44	

**Table 2:** Spectrum and frequency of skin disorders groups according to age groups (n = 6871)

Dermatoses	Infants	Preschool Age	School Age	Adolescent	Frequency n (%)
Infections	137	508	724	426	1795 (26.1)
Eczematous disorders	346	572	523	270	1711 (24.9)
Infestations	119	375	291	151	936 (13.6)
Papulosquamous eruptions	7	82	296	162	547 (8.0)
Bullous eruptions	16	42	131	65	254 (3.7)
Vitiligo	7	56	84	74	221 (3.2)
Acne vulgaris and acneiform eruptions	-	7	22	210	239 (3.5)
Urticaria and related disorders	7	21	66	103	197 (2.8)
Alopecia	5	18	59	43	125 (1.8)
Skin tumors	23	30	27	56	136 (2.0)
Naevi	10	31	24	26	91 (1.3)
Fixed drug eruptions	3	25	28	32	88 (1.3)
Nutritional dermatoses	3	24	46	7	80 (1.2)
Keloids and hypertrophic scars	1	25	28	34	88 (1.3)
Genetic and congenital abnormalities	2	18	20	21	61 (0.9)
Xerodermas	7	12	9	11	39 (0.6)
Connective tissue disorders	-	5	13	16	34 (0.5)
Granulomatous eruptions	4	10	17	1	32 (0.5)
Albinism	5	11	5	2	23 (0.3)
Pigmentary	2	5	9	9	25 (0.4)
Others	4	31	40	74	149 (2.2)

**Table 3:** Distribution of skin Infections and infestations (n=6871)*

Classification	Number	Percent
**Fungal infections**	**1,120**	16.3
Tinea capitis	554	8.1
Tinea pedis	385	5.6
Tinea corporis	282	4.1
Tinea manum	176	2.6
Tinea unguium	79	1.1
Tinea cruris	37	0.5
Tinea faciei	2	0.03
Tinea incognito	29	0.4
Pityriasis versicolor	253	3.7
Candidal Infections	114	1.7
Deep mycoses	4	0.1
**Viral**	**413**	6.0
Verrucae vulgaris	190	2.8
Molluscum contagiosum	141	2.1
Epidemodysplasia verruciformis	59	0.9
Viral exanthema	9	0.1
Herpes zoster	7	0.1
Herpes simplex	2	0.03
Eczema herpeticum	3	0.04
Chicken pox	2	0.03
**Bacteria**	**263**	3.8
Impetigo contagiosum	89	1.3
Mycobacteria infection	59	0.9
Periporitis	70	1.0
Furunculosis	20	0.3
Ecthyma	7	0.1
Cellulitis	3	0.04
Others	5	0.07
Unspecified	8	0.1
**Infestations**	**936**	13.6
Papular urticaria	705	10.2
Scabies	111	1.6
Onchodermatitis	106	1.5
Pediculosis capitis	2	0.03
Dermodex folliculitis	7	0.1
Others	3	0.04

^+^In this table some children had different types of both dermatophyte and yeast infections; dermatophyte infections occurred in multiple sites on the body concurrently Bacterial infections: others include Erysipelas; Erythrasma; Pitted keratolysis and Trichomycosis axillaris. Infestations: others include Myasis and osteomyelitis

**Table 4:** Frequency and spectrum of eczemas, xerodermas, pruritus, connective tissue, genetic, congenital disorders and related disorders

Classification	Number	Percent
**Eczemas**	**1,711**	**24.9**
Atopic dermatitis	1,042	15.1
Contact dermatitis	170	2.5
Seborrhoeic dermatitis	166	2.4
Erythroderma	77	1.1
Follicular eruptions	52	0.8
Pityriasis alba	32	0.5
Sycosis cruris	30	0.5
Gianotti Crosti syndrome	22	0.3
Hand dermatitis	21	0.3
Nummular eczema	21	0.3
Lichen striatus	21	0.3
Perioral dermatitis	14	0.2
Pityriasis amiantacea	5	0.1
Chronic dermatitis (unspecified)	8	0.1
Keratosis pilaris	7	0.1
Others	15	0.2
Unspecified	8	0.1
**Xerodermas**	**39**	**0.6**
Ichthyosis	33	0.5
Xerosis	6	0.1
**Urticaria, Pruritus and related disorders**	**197**	**2.9**
Urticaria	112	1.6
Lichen simplex chronicus	40	0.6
Pruritic papular eruptions	22	0.3
Pruritus	18	0.3
Urticaria pigmentosa	2	0.02
Mastocytosis	3	0.04
**Connective Tissue Disorder**	**34**	**0.5**
Systemic Lupus Erythematosus	11	0.2
Morphea	8	0.1
Rheumatoid Arthritis	3	0.04
Scleroderma	4	0.05
Discoid Lupus Erythematosus	3	0.04
Others	5	0.07
**Genetic And Congenital Disorders**	**61**	**0.9**
Neurofibromatoses	36	0.5
Tuberous Sclerosis	16	0.2
Congenital icthyosis	4	0.1
Others	5	0.1

Connective Tissue Disorders: others include Acute Cutaneous Lupus; Raynaud’s Phenomenon, Dermatomyositis, Juvenile idiopathic arthritis and Vasculitis; Genetic And Congenital Disorders others include Sturge Weber Syndrome; Aplasia Cutis, Piebaldism and Palmoplantar keratodema

**Table 5:** Frequency of papulosquamous eruptions, alopecias, granulomatous eruptions, skin tumors and bullous disorders

Classification	Number	Percent
**Papulosquamous eruptions**	**547**	**8.0**
Pityriasis rosea	237	3.4
Psoriasis	103	1.5
Pityriasis lichenoides chronica	38	0.6
Pityriasis lichenoides et varioliformis acuta	3	0.04
Pityriasis rubra pilaris	2	0.02
Lichenoid eruptions:	164	2.38
-Lichen planus	94	1.7
-Lichen nitidus	43	0.6
-Lichenoid drug eruption	5	0.07
-Lichen amyloid	1	0.01
**Bullous eruptions**	**254**	**3.7**
Childhood bullous eruption	221	3.2
Immunobullous disorders	12	0.17
Epidemolysis bullosa	8	0.12
Erythema multiforme	3	0.04
Dermatitis herpetiformis	3	0.04
Others	4	0.06
**Alopecia**	**125**	**1.8**
Alopecia Areata	101	1.45
Trichotillomania	4	0.06
Folliculitis Decalvans	5	0.07
Acne Folliculitis Nuchae	5	0.07
Dissecting Folliculitis.	3	0.04
Others	7	0.1
**Granulomatous Eruptions**	**32**	**0.47**
Granuloma Annulare	29	0.42
Erythema nodosum	2	0.03
Sarcoidosis	1	0.02
**Skin Tumors**	**136**	**1.98**
**Benign**	**128**	**1.86**
Haemangioma	37	0.5
Milaria	31	0.45
Syringoma	14	0.2
Pyogenic Granuloma	14	0.2
Actinic Keratosis	3	0.04
Papiloma	3	0.04
Trichoepithelioma	2	0.03
Others	24	0.35
**Malignant**	**8**	**0.12**
Kaposi’s Sarcoma	5	0.07
Others	3	0.04

Bullous eruptions: others include Grover’s disease, Collodion baby, Congenital ichthyosiform erythroderma Alopecia: others include Androgenetic Alopecia; Unclassified Scarring Alopecia; Non-Scarring Alopecia Benign skin tumors: others include Xanthoma, Angiokeratoma Circumscriptum, Calcinosis Cutis, Lymphangioma, Milia, Ganglion, Fibroma, Acrocordion, Chondrodermatitis Helicis, Histiocytoma, Bartholin’s Cyst and Seborrhoeic Keratosis, steacytoma and callus. Malignant skin tumors: others include Sezary Syndrome; Osteosarcoma, Lymphoproliferative Disorder

## Discussion

This study revealed that 23.6% of patients seen at the general dermatology clinic were of the pediatric age group. A wide range of skin disorders were seen in the study population groups similar to findings in previous surveys of outpatient clinics in Nigeria [14-16]. In addition, 8.4% of subjects had more than one diagnosis. This is comparable to studies by Hon et al [17] in China and Katibi et al [18] in South Africa who reported 11.1% and 9.8% respectively. According to Katibi et al [18] the presence of multiple skin conditions in some individuals draws attention to the need for thorough skin assessment of children to avoid missed diagnoses. The most frequently observed skin conditions in descending order were infections, eczemas, infestations, papulosquamous eruptions. This is in agreement with studies in community and hospital-based studies in Nigeria [4, 8-11]. A similar trend has been reported in India [19], but is in contrast to studies in developed countries such as Spain [20] and the United states [21] which reported a higher proportion of eczematous disorders. Infection was the most common group of skin disorders and the highest frequency was observed among the pre-school age and school age children. Tinea capitis (8.1%) was the most common skin infection A similar observation was made by Kiprono et al [22] in Tanzania who reported 19.1%. This observation is in contrast to 2.1% reported by Katibi et al [18] in South Africa. Tinea capitis is contagious, can be spread by sharing of personal items such as combs and shavers [23]. This condition is thought to be common in pre-adolescent children because of the absence of *Pityrosporum ovale*, a normal commensal and fungistatic fatty acids which are present in post-pubertal sebum [23]. Fungal skin infections flourish in areas of high humidity, poor personal hygiene and sanitation. As such there is a need to strengthen health education, personal hygiene and sanitation particularly among caregivers of younger children to control the spread of the infection which is known to occur in epidemics in schools [7,10]. Viral skin infections were the second most common cause of infections which mirrors a similar trend reported by Atraide et al [11] in Port-Harcourt, Nigeria but contrasts the study in South Africa [18] where viral warts accounted for 11% of diagnoses and was the most common skin infection. The high prevalence of HIV infection (almost 50% of the sample population) may explain the higher prevalence of viral warts in the South African study population. Although not life-threatening, viral warts should be recognized among primary care providers as a possible sign of immunosuppression requiring further evaluation particularly if the lesions are extensive and generalized.

Scabies (1.6%) was low in the index study, when compared to observations in hospital-based study in Tanzania (7.4%) [22] but higher than 0.7% reported in South Africa [18]. The low frequency of scabies may reflect management of cases at the primary level reducing the need for referral to the tertiary center. Papular urticaria (insect bite reactions) contributed 10.2% which was higher than observations in India (5.2%) [24] and Tanzania(5.6%) [22]. The high proportion observed could be explained by an interaction between the environment and the functional immaturity of the skin.[11] Papular urticaria affects children predominantly resulting from exaggerated response to insect bites and stings in the tropics when insect population is increased. In addition, urban areas with poor environmental sanitation and inadequate drainage may provide breeding areas for the biting insects like fleas and mosquitoes. Eczematous disorders (24.9%) were the second most frequently encountered category of dermatoses, with the highest number observed among infants (<2years) and pre-school age children. Atopic dermatitis (15.1%) was the most common eczematous disorder. This is comparable to observations by Atraide et al [11] and Emodi et al [25] who reported prevalence rates of 15.4% and 13.2% in Nigeria, and is close to prevalence rates reported in developed countries like United states [21] and Denmark [26]. The high prevalence of atopic dermatitis in the index study may be due to referral of cases to the specialty clinic. Over the last four decades, a four-fold increase in the prevalence of atopic dermatitis has been reported worldwide due to rural-urban migration and adoption of western lifestyles [14 ,27]. A similar trend has been demonstrated in Nigeria as a prevalence rate of 2.6% was reported in 1989 [28]. . In addition, Atopic dermatitis is associated with quality of life issues such as poor school performance and poor sleep. Therefore, there is a need to train primary care physicians on recognition of symptoms and signs to ensure early diagnosis and treatment to reduce the growing disease burden. Acne vulgaris was found most predominantly in the adolescent age due to high production of sebum and androgens associated with puberty [29]. Adolescence is characterized by adjustment to psychological, social and physical changes. The presence of severe acne vulgaris during such a period can have significant effect on the psychological wellbeing and quality of life of affected individuals in this age group [30, 31]. Impaired peer relationships and poor school performance have been reported in affected individuals in developed countries [30]. As such, primary care physicians and general paediatricians need to be made aware of the importance of prompt diagnosis, treatment and referral of severe cases so as to ameliorate impact of Acne on quality of life. Being a hospital-based study, the results of this study should be interpreted cautiously as it may not be fully representative of the prevalence of paediatric dermatological conditions in the general population. A larger prospective study involving both the community and outpatient clinics may be more encompassing. In light of the current pattern of paediatric dermatoses demonstrated in this study it is necessary to raise awareness among primary care physicians and every healthcare worker on prompt diagnosis and treatment of common skin conditions. Monroe et al [32] noted that in developing countries non-dermatologists often miss common diagnoses leading to inappropriate diagnosis and treatment. A similar observation was made by Emodi et al [25] in a study of children in Enugu. Therefore, in agreement with the WHO campaign for strengthening community dermatology services [33], there is a need to improve the dermatology curriculum at the basic and specialist training levels.

## Conclusion

In conclusion, the study shows that infections such as tinea capitis and viral warts are still very common among children attending the dermatology clinic. However, the prevalence of atopic dermatitis may be on the increase. With the wide spectrum of diseases reported and limited number of trained specialists, additional training of paediatric residents and primary care physicians will significantly improve first-line treatment outcomes reducing inaccurate treatment. This study provides elaborate information for future epidemiological and clinical research. It might also help to assess the changing trends of pediatric dermatoses.

### What is known about this topic

- Community-based studies in Nigeria have reported a high incidence of dermatophytosis and viral skin infections in children;
- Hospital-based studies report a high incidence of eczematous disorders among infants.

### What this study adds

- In the study the most common category of skin diseases were infections, eczemas, infestations, papulosquamous eruptions;
- The study shows that infections such as tinea capitis and viral warts are still very common among children attending the dermatology clinic;
- The high prevalence of eczematous disorders such as atopic dermatitis reported in the study suggests the prevalence of this condition may be on the increase.
